# Molecular characterization of mupirocin-resistant MRSA from Germany and South Africa

**DOI:** 10.3389/fcimb.2026.1865925

**Published:** 2026-08-11

**Authors:** Adebayo Osagie Shittu, Olga Perovic, Franziska Layer-Nicolaou, Birgit Strommenger, Tomiwa Olumide Adesoji, Taofiq Adewale Sulayman, Ayorinde Oluwatobiloba Afolayan, Alexander Mellmann, Frieder Schaumburg

**Affiliations:** 1 Institute of Medical Microbiology, University Hospital Münster, Münster, Germany; 2 Centre for Healthcare-Associated Infections, Antimicrobial Resistance and Mycoses (CHARM) at National Institute for Communicable Diseases, a division of National Health Laboratory Service (NHLS), and University of Witwatersrand, Johannesburg, South Africa; 3 National Reference Centre (NRC) for Staphylococci and Enterococci, Division of Nosocomial Pathogens and Antibiotic Resistances, Department of Infectious Diseases, Robert Koch Institute, Wernigerode, Germany; 4 Department of Microbiology, Obafemi Awolowo University, Ile-Ife, Nigeria; 5 Leibniz Institute-German Collection of Microorganisms and Cell Cultures, Braunschweig, Germany; 6 Institute for Hygiene, University Hospital Münster, Münster, Germany

**Keywords:** Germany, high-level mupirocin resistance, low-level mupirocin resistance, *mupA*-plasmids, mupirocin-resistant methicillin-resistant *Staphylococcus aureus*, South Africa

## Abstract

**Introduction:**

Methicillin-resistant *Staphylococcus aureus* (MRSA) is a human pathogen of global public health importance. A key element of MRSA decolonization strategies is the administration of mupirocin, an antibiotic also used to treat superficial skin infections. However, the emergence and global increase of mupirocin-resistant (mupR)-MRSA highlight the need for active surveillance to support evidence-based MRSA control measures. This study investigated the antibiotic resistance, genetic diversity, and plasmid profiles of mupR-MRSA from Germany and South Africa.

**Methods:**

Phenotypic identification of mupR-MRSA strains was verified by molecular methods. Characterization of all strains included PCR detection of Panton-Valentine leucocidin, immune evasion cluster genes, and staphylococcal protein A typing. Representative strains from each spa type were selected for whole-genome sequencing to determine their clonal lineages and relationships, including antibiotic and virulence gene content. Comparative analysis, phylogeny and classification of *mupA*-carrying plasmids were determined.

**Results:**

Eighty-two mupR-MRSA were characterized, comprising 32 strains that exhibited high-level mupirocin resistance (HmupR) and 50 strains with low-level mupirocin resistance (LmupR). The plasmid-mediated *mupA* gene and the V588F mutation in the bacterial isoleucyl-tRNA synthetase gene were identified in strains that demonstrated HmupR and LmupR, respectively. However, *mupA* and the V588F mutation were detected in two strains from Germany. The mupR-MRSA from Germany were assigned to CC1, CC5, CC8, CC22, CC30, and ST59, while those from South Africa were grouped into CC8, CC22, and CC30. The strains harboured different SCC*mec* types (IIa, III, IVa, IVj, and V). Notably, mupR CC22-MRSA-IVj and tst-positive CC30-MRSA-IIa strains were identified in both countries. The *mupA*-positive plasmids formed four distinct communities, including two singletons. The *mupA*-positive plasmids from the strains in Germany often carried the aminoglycoside (*aac(6')-Ie/aph(2'')-Ia*) resistance genes. However, the β-lactam (*blaZ*), arsenic (*arsB, arsC, arsR*), and copper (*mco*) resistance genes were primarily identified on *mupA*-positive plasmids from strains obtained in South Africa.

**Discussion:**

This study highlights the genetic diversity and potential for horizontal gene transfer among *mupA*-positive MRSA lineages in the two countries, further supported by the regional clustering of *mupA* plasmids. Integrating genomic surveillance of chromosomal- and plasmid-mediated resistance with antimicrobial use and clinical data is needed for targeted MRSA infection prevention and control.

## 1 Introduction

Methicillin-resistant *Staphylococcus aureus* (MRSA) infections are associated with high morbidity and mortality rates, as well as high economic costs (Zhen et al., 2020). One of the primary strategies to minimize MRSA infections among patients and healthcare workers is intranasal mupirocin decolonization (Hetem et al., 2016). Mupirocin is a naturally occurring antibiotic that interferes with bacterial protein synthesis (Fuller et al., 1971). However, reports of mupirocin-resistant (mupR)-MRSA have emerged, and a global increase in its prevalence has been observed (Dadashi et al., 2020). This phenomenon is largely associated with unrestricted and long-term use of mupirocin in healthcare settings (Hetem et al., 2016). MupR-MRSA turns decolonization in colonized patients and personnel ineffective (Hetem and Bonten, 2013). Moreover, mupirocin resistance may also promote the spread of multidrug resistance through co-selection with other antibiotic resistance genes (Carter et al., 2018).

Two levels of mupirocin resistance have been described: low-level mupirocin resistance (LmupR with a minimum inhibitory concentration [MIC] of 8–256 µg/ml) and high-level mupirocin resistance (HmupR with an MIC of ≥512 µg/ml) (Patel et al., 2009). LmupR is caused by chromosomal mutations (primarily V588F and V631F) in the bacterial isoleucyl-tRNA synthetase (*ileS*) gene (Antonio et al., 2002). HmupR is mediated by plasmids carrying *mupA*, which encodes an alternative isoleucyl-tRNA synthetase (*ileS2*) gene with very low affinity for mupirocin (Gilbart et al., 1993). Another determinant of HmupR (mupB, MIC ≥1,024 µg/ml) has also been described. It showed 65.5% and 45.5% sequence identity with *mupA* and *ileS*, respectively (Seah et al., 2012).

Data from systematic reviews have shown that the prevalence of mupR *S. aureus* in Africa and mupR-MRSA in Europe is above 10% (Shittu et al., 2018; Dadashi et al., 2020). However, information on the regional and global epidemiology of mupR-MRSA remains limited. Addressing this knowledge gap is vital, as the rising global prevalence of mupR-MRSA underscores the need for active surveillance and characterization to support effective infection prevention and control. This study examined the antibiotic resistance, genetic determinants, clonal lineages, and plasmid landscape of mupR-MRSA in two continents: Africa (South Africa) and Europe (Germany).

## 2 Materials and methods

### 2.1 Collection of strains and confirmation as *S. aureus* and MRSA

MupR-MRSA strains from Germany (n=42, 2018-2020) and South Africa (n=40, 2012-2017) were evaluated. The German collection comprised strains (n=37) from local microbiological laboratories, which were forwarded to the National Reference Centre for Staphylococci and Enterococci at the Robert Koch Institute in Wernigerode for characterization. MupR-MRSA (n=5) from clinical samples at the Institute of Medical Microbiology, Universitätsklinikum Münster (UKM), Germany, were also included in this study. The South African collection included mupR-MRSA from a study conducted by the Group for Enteric, Respiratory, and Meningeal Surveillance in South Africa (GERMS-SA) (Fortuin-de Smidt et al., 2015; Perovic et al., 2017).

Phenotypic identification as *S. aureus* was substantiated by MALDI-TOF MS (Sirius One, Bruker Daltonics, Bremen, Germany). DNA extraction was performed using the QIAamp tissue kit according to the manufacturer’s instructions (QIAGEN GmbH, Hilden, Germany), with a slight modification (the addition of lysostaphin at the lysing step) for Gram-positive cocci. The strains were confirmed as *S. aureus* by PCR detection of the species-specific thermonuclease (*nuc*) (Brakstad et al., 1992) and the non-ribosomal peptide synthetase (NRPS) genes (Zhang et al., 2016). PCR detection of the *mecA* gene (Bignardi et al., 1996) verified the strains as MRSA.

### 2.2 Antibiotic susceptibility testing and identification of strains exhibiting LmupR and HmupR

The mupR-MRSA strains were subjected to antibiotic susceptibility testing using the VITEK 2 automated system (bioMérieux, Marcy l’Ètoile, France) and the EUCAST clinical breakpoints (Version 12). Mupirocin MIC was measured using VITEK for screening and a gradient-diffusion test for confirmation (E-test, bioMérieux; range: 0.064-1024 µg/ml). Mupirocin resistance was defined as described previously (Patel et al., 2009). Furthermore, HmupR was confirmed by PCR detection of *mupA* (Shittu and Lin, 2006).

#### 2.2.1 Statistical analysis

Proportions (e.g. antibiotic resistance rates) of mupR-MRSA from Germany and South Africa were compared using Fisher’s exact test, and p-values of <0.05 were considered statistically significant.

### 2.3 Molecular characterization of mupR-MRSA and whole-genome sequencing of representative strains

Characterization of the mupR-MRSA strains included PCR detection of the *lukS*-PV/*lukF*-PV (Lina et al., 1999) and of the immune evasion cluster (IEC: *chp*, *sak*, *scn*) genes (Van Wamel et al., 2006). Moreover, staphylococcal protein A (*spa*) typing (Harmsen et al., 2003) was conducted, and one representative strain from each *spa* type (from both countries) was selected for whole-genome sequencing (WGS) to determine their population structure, including antibiotic resistance, virulence, and plasmid gene content.

For WGS, DNA was isolated using the NEB Monarch Genomic Purification Kit (New England Biolabs, Ipswich, MA, USA). WGS was performed on a PacBio^®^ Sequel IIe system (Pacific Biosciences, Menlo Park, CA, USA) using the SMRTbell^®^ Express Template Prep Kit 2.0 (Pacific Biosciences Inc.). PacBio sequencing data were assembled *de novo* using the SMRT^®^ Link software suite v10 with default parameters. Strain sequences were annotated using Prokka (1.14.6) on the Galaxy platform (Seemann, 2014). Antimicrobial resistance, including biocide, heavy-metal resistance and virulence genes, was predicted *in silico* with the AMRFinder Plus (v3.12.8, database 2024-07-22.1) command-line tool (Feldgarden et al., 2021). The staphylococcal cassette chromosome *mec* (SCC*mec*) types (and subtypes) of the strains were determined using the Centre for Genomic Epidemiology tools, SCC*mec*Finder 1.2 (Camacho et al., 2009; Kaya et al., 2018) and SCC*mec* (https://github.com/rpetit3/sccmec), a tool for typing SCC*mec* cassettes in assemblies (Petit, 2024). The sequence types (STs) were determined by multilocus sequence typing (MLST) using SeqSphere+ (v9).

A pan-genome was constructed using Roary (v. 3.13.0) (Page et al., 2015) to identify core genes shared across all strains. The alignment of core genes (present in 99% of strains) was generated using default parameters, including a minimum percentage identity of 95% for clustering homologous genes. Core gene sequences were aligned with MAFFT (7.526) (Katoh and Standley, 2013) using the auto-selected strategy. A maximum-likelihood phylogeny was inferred from the untrimmed alignment using IQ-Tree (v. 2.4.0) (Nguyen et al., 2014) under the general time reversible substitution model with 1,000 ultrafast bootstrap replicates for branch and node support. The phylogenetic tree was visualized and annotated using iTOL (v6.7.4) (Letunic and Bork, 2021). The epidemiological, phenotypic, and gene-content data for the mupR-MRSA strains were mapped onto the phylogenetic tree to facilitate comparative phylogenomic analysis. All analyses were conducted on the public Galaxy server (https://usegalaxy.org). Genome sequences of the representative strains were deposited on the NCBI website under Bioproject accession number PRJNA1060627.

### 2.4 Phylogenetic contextualization of ST8 and ST22 strains

To contextualize the ST8 and ST22 strains in this study within the USA300 and EMRSA-15 lineages, sequence reads and associated metadata from previous studies, i.e., for ST8 (Virgillio et al., 2025), and ST22 (Holden et al., 2013) were retrieved from the NCBI Sequence Read Archive (SRA) (ST8) or the European Nucleotide Archive (ST22) using fastq-dl (v4.0.1) (Petit, 2019). Reads were assembled using Shovill (v1.4.2) (https://github.com/tseemann/shovill (Accessed June 1, 2026), and empty assemblies were excluded. Sequence types were verified using the MLST tool (v2.33.1) (https://github.com/tseemann/mlst (Accessed June 18, 2026), and assemblies not assigned to sequence types ST8 or ST22 were excluded from downstream analyses.

A total of 176 ST8 and 245 ST22 genome assemblies were annotated using Bakta (v1.12.0) (Schwengers et al., 2021) with the Bakta database (light, v6.0). For each sequence type, filtered core-genome alignments were generated from the annotation files using Panaroo (v1.7.0) (Tonkin-Hill et al., 2020). Maximum-likelihood phylogenies were reconstructed from the filtered core genome alignments using IQ-TREE (v3.1.2) (Wong et al., 2026) and visualized in iTOLv7 (Letunic and Bork, 2024). Lineage assignments of the ST8 and CC22 genomes were independently verified using the lineage module of Staphscope (v1.2.2) (Beckley and Amarh, 2026).

### 2.5 Structural comparison of the SCC*mec* elements

SCC*mec*-associated genes and structural components were identified from genome annotation files generated for strains in this study. Complete SCC*mec* elements were extracted from chromosomal assemblies using the subseq module of SeqKit (v2.10.1) (Shen et al., 2024). Accession numbers of reference SCC*mec* elements were retrieved from a previous study (Urushibara et al., 2020) and the SCC*mec* database (Petit, 2024; https://github.com/rpetit3/sccmec/blob/main/data/sccmec-regions.tsv). To facilitate comparative analyses, both the extracted SCC*mec* sequences and reference elements were annotated using Bakta (Schwengers et al., 2021). Structural similarity and synteny among SCC*mec* elements were assessed using all-versus-all sequence alignments generated with Minimap2 (v2.30-r1287) (Li, 2018). Alignment results, together with the corresponding GFF3 annotations files, were visualized using the genomes R package (v1.1.3) (Hackl et al., 2024) to compare the genetic organization and structural conservation of SCC*mec* elements.

### 2.6 Comparative analysis, phylogeny and classification of *mupA*-carrying plasmids

Putative plasmid sequences were identified from long-read sequence data using Plasflow (v1.1) (Krawczyk et al., 2018). This machine-learning tool classified contigs as plasmids or chromosomal based on k-mer composition. Identified plasmids were annotated using Bakta (v1.11.3) along with the Bakta reference database (v6.0.0) (Schwengers et al., 2021; Beyvers et al., 2025). The reference plasmid (*S. aureus* pPR9 - GenBank accession number GU237136) was processed by extracting all annotated coding sequences (CDSs) using gffread (v2.2.1), and the resulting FASTA file was formatted into a nucleotide BLAST database using NCBI BLAST+ (makeblastdb). CDS sequences from seventeen query plasmids in this study, also extracted with gffread, were aligned against the reference database using BLASTN in megablast mode. For each plasmid gene, the highest-scoring BLAST hit was retained as its closest reference match. Gene names and product descriptions for the reference plasmid were parsed from its GenBank file and merged with the BLAST output to annotate each match. A gene was considered present in a plasmid if it showed at least 80% identity and 80% alignment coverage with the corresponding reference gene. These matches were used to build a gene presence/absence matrix and to calculate plasmid similarity scores based on the proportion of reference genes detected in each plasmid. All data processing, merging, and summary calculations were performed using custom Python scripts built around the pandas library. BRIG (v0.95) (Alikhan et al., 2011) was used for plasmid visualization and comparison.

Identified *mupA*-positive contigs were extracted using the publicly available Perl script extract-contig.pl (https://github.com/raymondkiu/bioinformatics-tools/blob/master/extract-contig.pl). Pairwise Mash distances were calculated with Mashtree (v1.4.6) (Katz et al., 2019) using a threshold of 0.001 to define highly similar plasmids, as previously described (Roberts et al., 2023; Akintayo et al., 2025), and a neighbor-joining phylogenetic tree was generated.

To evaluate the relationship between *mupA*-positive plasmids identified in ST8 strains in this study and previously described USA300-associated *mupA*-carrying plasmids, reference sequences (NZ_CP136525.1, NZ_CP136529.1, and NC_007792.1) were retrieved from the plasmid database PLSDB (Galata et al., 2019; Schmartz et al., 2022; Molano et al., 2025). Pairwise Mash distances and a neighbor-joining phylogeny were generated to assess their genetic similarity and clustering patterns.

Plasmid synteny was assessed by performing all-versus-all sequence alignments with Minimap2 (v2.30-r1287) (Li, 2018). Alignment results, together with annotations (GFF3 format), were visualized using the gggenomes R package (v1.1.3) (Hackl et al., 2024) to compare the genetic architecture of *mupA*-carrying plasmids.

Plasmid mobilization genes and mobility types were predicted with Mobtyper (v3.1.9) (Robertson and Nash, 2018). The relaxase mobilization (MOB) families of the *mupA*-carrying plasmids were determined using MOBSCAN (Garcillán-Barcia et al., 2019). Plasmid replication (Rep) groups were assigned using Staphscope (v1.2.2) (Beckley and Amarh, 2026), which utilizes Abricate (v1.4.0) (Seemann, 2014) and the PlasmidFinder database (2026-April-3) (Carattoli et al., 2014).

To classify conjugative plasmids into established staphylococcal plasmid families, a custom BLAST database was constructed from representative reference plasmids of the pGO1 (FM207042.1), pSK41 (AF051917.1), and pWBG749 (GQ900391.1) families using the NCBI BLAST+ suite (makeblastdb). Conjugative plasmids identified in this study were queried against this database using blastn, and family assignments were made based on matches satisfying the following thresholds: nucleotide identity >99%, query coverage ≥50%, and bitscore >20,000.

Community networks of *mupA*-carrying plasmids were built using Pling (v2.0.0, default settings) (Frolova et al., 2024) and visualized in Cytoscape (v3.10.3) (Shannon et al., 2003) via RCy3 (v2.28.1) (Gustavsen et al., 2019). iTOL annotation files were generated with itol.toolkit (v1.1.7) (Zhou et al., 2023). All network and annotation processing were performed in R (v4.5.1). Phylogenetic trees and metadata for *mupA*-carrying contigs were visualized in iTOL (Letunic and Bork, 2021).

## 3 Results

### 3.1 Antibiotic susceptibility testing of mupR-MRSA strains

All the mupR-MRSA from Germany (n=42) and South Africa (n=40) were *mecA*-positive and susceptible to linezolid, tigecycline, and vancomycin (**Table 1**, **Supplementary Table 2**). However, 98% of the strains were resistant to ciprofloxacin, 74% to erythromycin, 72% to clindamycin, and 45% to gentamicin (**Table 1**). The mupR-MRSA from Germany showed significantly lower resistance rates compared to those from South Africa to clindamycin (inducible resistance, 10% vs. 60%, OR 0.07, p<0.0001), tetracycline (7% vs. 53%, OR 0.07, p<0.0001), trimethoprim-sulfamethoxazole (2% vs. 50%, OR 0.02, p<0.0001) and erythromycin (64% vs. 85%, OR 0.32, p=0.043, **Table 1**). No significant differences were observed for the remaining antimicrobials.

**Table 1:** Antibiotic resistance rates of low- and high-level mupR-MRSA from Germany and South Africa.

Antimicrobial agent	Resistance rates % (n)			Resistance rates % (n)			OR (Germany vs South Africa)	95% CI	P-value
	Germany			South Africa					
	LmupR	HmupR	Total	LmupR	HmupR	Total			
	(n=23)	(n=19)	(n=42)	(n=27)	(n=13)	(n=40)			
Ciprofloxacin	100% (23)	90% (17)	95% (40)	100% (27)	100% (13)	100% (40)	0.20	0.01–4.30	0.494
Clindamycin	65% (15)	63% (12)	64% (27)	100% (27)	39% (5)	80% (32)	0.45	0.17–1.22	0.143
Inducible clindamycin resistance	13% (3)	5% (1)	10% (4)	70% (19)	39% (5)	60% (24)	0.07	0.02–0.24	<0.0001
Erythromycin	65% (15)	63% (12)	64 (27)	100% (27)	54% (7)	85% (34)	0.32	0.11–0.93	0.043
Fusidic acid	4% (1)	16% (3)	10% (4)	0% (0)	15% (2)	5% (2)	2.00	0.35–11.58	0.676
Gentamicin	4% (1)	84% (16)	40% (17)	70% (19)	8% (1)	50% (20)	0.68	0.28–1.63	0.506
Linezolid	0% (0)	0% (0)	0% (0)	0% (0)	0% (0)	0% (0)	NA	NA	1.00
Rifampicin	4% (1)	11% (2)	7% (3)	4% (1)	8% (1)	5% (2)	1.46	0.23–9.24	1.00
Tetracycline	4% (1)	11% (2)	7% (3)	70% (19)	15% (2)	53% (21)	0.07	0.02-0.26	<0.0001
Tigecycline	0% (0)	0% (0)	0% (0)	0% (0)	0% (0)	0% (0)	NA	NA	1.00
Trimethoprim-sulfamethoxazole	0% (0)	5% (1)	2% (1)	70% (19)	8% (1)	50% (20)	0.02	0.003-0.19	<0.0001
Vancomycin	0% (0)	0% (0)	0% (0)	0% (0)	0% (0)	0% (0)	NA	NA	1.00

LmupR, low-level mupirocin resistance; HmupR, high-level mupirocin resistance, OR, Odds Ratio; CI, Confidence Interval.

Based on the E-test results, 32 strains exhibited HmupR, and 50 strains demonstrated LmupR (**Table 1**). All the mupR-MRSA with an E-test MIC of ≥1024µg/ml were *mupA*-positive. A high degree of essential agreement (98%; 80/82) was observed between the VITEK and E-test methods in identifying mupR-MRSA exhibiting LmupR and HmupR. In one result not within essential agreement, the VITEK MIC was 2µg/ml, whereas the E-test MIC was 12µg/ml. In the second result, the E-test MIC was 24µg/ml, whereas the VITEK MIC was >512µg/ml (**Table 2**).

**Table 2:** Antibiotic susceptibility testing and genotyping of mupR-MRSA from Germany.

									PCR			WGS
Strain number	Spa type	ST	CC	Antimicrobial resistance	VITEK µg/ml	E-test µg/ml	*mupA*	*lukS PV/lukF-PV*	*chp*	*sak*	*scn*	*mupA*/*V588F*
1800310	t127	ST1	CC1	FUS, MUP(H)	>512	>1024	+	+	–	+	+	*mupA*
1900281	t045	ST5	CC5	CIP, ERY, MUP(L)	32	32	–	–	+	+	+	ND
1903807	t045	ST5	CC5	CIP, ERY, GEN, MUP(H)	>512	>1024	+	–	+	+	+	*mupA*
1900528	t062	ST5	CC5	CIP, ERY, MUP(L)	32	32	–	–	+	+	+	V588F
1801526	t003	ST225	CC5	CIP, ERY, GEN, MUP(H)	>512	>1024	+	–	+	+	+	ND
1801538	t003	ST225	CC5	CIP, ERY, MUP(L)	32	32	–	–	+	+	+	V588F
1900607	t003	ST225	CC5	CIP, ERY, MUP(L)	32	24	–	–	+	+	+	ND
1901009	t003	ST225	CC5	CIP, ERY, MUP(L)	32	24	–	–	+	+	+	ND
71614677	t1107	ST225	CC5	CIP, ERY, MUP(H)	>512	24	–	–	+	+	+	V588F
1900632	t008	ST8	CC8	CIP, MUP(L)	32	24	–	–	–	+	+	V588F
1900696	t008	ST8	CC8	CIP, ERY, MUP(L)	32	12	–	–	–	+	+	ND
71654646	t008	ST8	CC8	CIP, ERY, MUP(L)	32	16	–	–	–	+	+	ND
1903798	t1476	ST8	CC8	CIP, COT, ERY, GEN, MUP(H), TET	>512	>1024	+	–	+	+	+	*mupA*
1801878	t030	ST239	CC8	CIP, ERY, GEN, MUP(L), RIP, TET	32	32	–	–	–	–	–	V588F
1901905	t022	ST22	CC22	CIP, ERY, MUP(L)	32	32	–	–	+	+	+	V588F
1902307	t022	ST22	CC22	CIP, MUP(L)	32	32	–	–	+	+	+	ND
17710027703	t022	ST22	CC22	CIP, ERY, FUS, GEN, MUP(H)	>512	>1024	+	–	–	–	–	ND
1800007	t032	ST22	CC22	CIP, ERY, GEN, MUP(H)	>512	>1024	+	–	+	+	+	ND
1800139	t032	ST22	CC22	CIP, MUP(L)	32	16	–	–	+	+	+	ND
1800214	t032	ST22	CC22	CIP, ERY, GEN, MUP(H)	>512	>1024	+	–	+	+	+	ND
1800389	t032	ST22	CC22	CIP, MUP(L)	32	32	–	–	–	–	–	ND
1800559	t032	ST22	CC22	CIP, ERY, GEN, MUP(H), TET	>512	>1024	+	–	+	+	+	ND
1800785	t032	ST22	CC22	CIP, ERY, MUP(H)	>512	>1024	+	–	+	+	+	ND
1801044	t032	ST22	CC22	CIP, ERY, GEN, MUP(H)	>512	>1024	+	–	+	+	+	ND
1801357	t032	ST22	CC22	CIP, ERY, MUP(L)	32	48	–	–	+	+	+	ND
1802001	t032	ST22	CC22	CIP, ERY, MUP(L)	32	32	–	–	+	+	+	V588F
1802319	t032	ST22	CC22	CIP, ERY, MUP(L)	32	12	–	–	–	–	–	ND
1802390	t032	ST22	CC22	CIP, GEN, MUP(H)	>512	>1024	+	–	–	–	–	ND
1900065	t032	ST22	CC22	CIP, ERY, GEN, MUP(H)	>512	>1024	+	–	–	–	–	ND
1900066	t032	ST22	CC22	CIP, MUP(L)	32	32	–	–	+	+	+	ND
1900147	t032	ST22	CC22	CIP, ERY, MUP(L)	32	16	–	–	+	+	+	ND
1901840	t032	ST22	CC22	CIP, GEN, MUP(H)	>512	>1024	+	–	+	+	+	ND
1902103	t032	ST22	CC22	CIP	2	12	–	–	–	–	–	ND
71640384	t032	ST22	CC22	CIP, ERY, FUS, MUP(L)	32	24	–	–	+	+	+	ND
1710022031	t032	ST22	CC22	CIP, ERY, GEN, MUP(H)	>512	>1024	+	–	–	+	+	ND
1800017	t492	ST22	CC22	CIP, MUP(L)	32	24	–	–	–	–	–	V588F
1802524	t1031	ST22	CC22	CIP, MUP(L)	32	16	–	–	+	+	+	V588F
1802291	n/a	ST22	CC22	CIP, GEN, MUP(H), RIP	>512	>1024	+	–	+	+	+	V588F/*mupA*
1802582	n/a	ST22	CC22	CIP, GEN, MUP(H), RIP	>512	>1024	+	–	+	+	+	V588F/*mupA*
1901548	t018	ST30	CC30	GEN, MUP(H)	>512	>1024	+	–	–	+	+	*mupA*
1909000	t021	ST36	CC30	CIP, ERY, MUP(H)	>512	>1024	+	–	+	+	+	*mupA*
1902157	t172	ST59	**-**	CIP, FUS, GEN, MUP(H)	>1024	>1024	+	–	+	+	+	*mupA*

+, Detected; -, Not detected; ND, Not determined.

### 3.2 Clonal lineages of mupR-MRSA from Germany and South Africa

The mupR-MRSA from Germany were categorized into 15 *spa* types and distributed across five clonal complexes (CCs): CC1, CC5, CC8, CC22, CC30, and one singleton: sequence type ST59 (**Table 2**). The *mupA* gene and the V588F mutation were identified in selected strains for WGS that demonstrated HmupR and LmupR, respectively. However, both the *mupA* and V588F mutations were detected in two CC22 strains from Germany. The mupR-MRSA from South Africa were grouped into 11 *spa* types and three CCs comprising CC8, CC22, and CC30 (**Table 3**). Eleven of the 13 *mupA*-positive MRSA from South Africa were classified in CC22, and LmupR (V588F mutation) was widespread across CC8 and CC30 (**Table 3**).

**Table 3:** Antibiotic susceptibility testing and genotyping of mupR-MRSA from South Africa.

									PCR			WGS
Strain number	Spa type	ST	CC	Antimicrobial resistance	VITEK µg/ml	E-test µg/ml	*mupA*	*lukS PV/lukF-PV*	*chp*	*sak*	*scn*	*mupA*/*V588F*
8227	t008	ST8	CC8	CIP, ERY, MUP(H)	>512	>1024	+	+	+	+	+	*mupA*
6248	t037	ST239	CC8	CIP, COT, ERY, GEN, MUP(L), TET	32	16	–	–	–	+	+	ND
7008	t037	ST239	CC8	CIP, COT, ERY, GEN, MUP(L), TET	32	16	–	–	–	+	+	ND
7120	t037	ST239	CC8	CIP, COT, ERY, GEN, MUP(L), TET	32	24	–	–	–	+	+	ND
7166A	t037	ST239	CC8	CIP, COT, ERY, GEN, MUP(L), TET	32	24	–	–	–	+	+	ND
7319	t037	ST239	CC8	CIP, COT, ERY, GEN, MUP(L), TET	32	24	–	–	–	+	+	ND
7341	t037	ST239	CC8	CIP, COT, ERY, GEN, MUP(L), TET	32	24	–	–	–	+	+	ND
7469	t037	ST239	CC8	CIP, COT, ERY, GEN, MUP(L), TET	32	24	–	–	–	+	+	V588F
7501	t037	ST239	CC8	CIP, COT, ERY, GEN, MUP(L), TET	32	16	–	–	–	+	+	ND
7529	t037	ST239	CC8	CIP, COT, ERY, GEN, MUP(L), TET	32	16	–	–	–	+	+	ND
7530	t037	ST239	CC8	CIP, COT, ERY, GEN, MUP(L), TET	32	24	–	–	–	+	+	ND
7547A	t037	ST239	CC8	CIP, COT, ERY, GEN, MUP(L), TET	32	24	–	–	–	+	+	ND
7610B	t037	ST239	CC8	CIP, COT, ERY, GEN, MUP(L), TET	32	24	–	–	–	+	+	ND
7792A	t037	ST239	CC8	CIP, COT, ERY, GEN, MUP(L), TET	32	32	–	–	–	+	+	ND
7821	t012	ST239	CC8	CIP, ERY, MUP(L)	32	32	–	–	+	+	+	ND
7826	t037	ST239	CC8	CIP, COT, ERY, GEN, MUP(L), TET	32	32	–	–	–	+	+	ND
7881A	t037	ST239	CC8	CIP, COT, ERY, GEN, MUP(L), TET	24	24	–	–	–	+	+	ND
8027A	t037	ST239	CC8	CIP, COT, ERY, GEN, MUP(L), TET	32	16	–	–	–	+	+	ND
9815A	t037	ST239	CC8	CIP, COT, ERY, GEN, MUP(L), TET	32	24	–	–	–	+	+	ND
10922B	t037	ST239	CC8	CIP, COT, ERY, GEN, MUP(L), TET	32	24	–	–	–	+	+	ND
7470A	n/a	ST239	CC8	CIP, COT, ERY, GEN, MUP(L), TET	32	16	–	–	–	+	+	V588F
6058	t1257	ST612	CC8	CIP, COT, ERY, GEN, MUP(H), RIP, TET	>512	>1024	+	–	–	+	+	*MupA*
8538B	t032	ST22	CC22	CIP, ERY, MUP(H)	>512	>1024	+	–	+	+	+	ND
8540	t032	ST22	CC22	CIP, FUS, MUP(H)	>512	>1024	+	–	+	+	+	ND
9210	t032	ST22	CC22	CIP, MUP(H)	>512	>1024	+	–	+	+	+	ND
9270	t032	ST22	CC22	CIP, MUP(H)	>512	>1024	+	–	+	+	+	ND
9876B	t032	ST22	CC22	CIP, FUS, MUP(H)	>512	>1024	+	–	–	–	–	ND
10486	t032	ST22	CC22	CIP, MUP(H)	>512	>1024	+	–	+	+	+	*MupA*
11758	t294	ST22	CC22	CIP, ERY, MUP(H)	>512	>1024	+	–	+	+	+	*MupA*
8695A	t1467	ST22	CC22	CIP, ERY, MUP(H)	>512	>1024	+	–	+	+	+	*MupA*
8866	n/a	ST22	CC22	CIP, ERY, MUP(H)	>512	>1024	+	–	–	–	–	*MupA*
9616	t1467	ST22	CC22	CIP, ERY, MUP(H)	>512	>1024	+	–	+	+	+	ND
11360	t20420	ST22	CC22	CIP, MUP(H), TET	>512	>1024	+	–	+	+	+	*MupA*
7514	t012	ST36	CC30	CIP, ERY, MUP(L), RIP	32	16	–	–	+	+	+	V588F
7863	t012	ST36	CC30	CIP, ERY, MUP(L)	32	24	–	–	+	+	+	ND
7880A	t012	ST36	CC30	CIP, ERY, MUP(L)	32	16	–	–	–	–	–	ND
7839	t018	ST36	CC30	CIP, ERY, MUP(L)	32	32	–	–	+	+	+	V588F
5978	t021	ST36	CC30	CIP, ERY, MUP(L)	32	16	–	–	+	+	+	V588F
7471	t021	ST36	CC30	CIP, ERY, MUP(L)	32	16	–	–	–	+	–	ND
7853	t238	ST36	CC30	CIP, ERY, MUP(L)	32	24	–	–	+	+	+	V588F

CIP, Ciprofloxacin; CLIN, Clindamycin; COT, Trimethoprim/Sulfamethoxazole; ERY, Erythromycin; FUS, Fusidic acid; GEN, Gentamicin; MUP(L), Mupirocin (low-level resistance); MUP(H), Mupirocin (high-level resistance); OXA, Oxacillin; RIP, Rifampicin; TET, Tetracycline. LukS-PV/LukF-PV, Subunit S and Subunit F; chp, chemotaxis inhibitory protein; sak, staphylokinase; scn, staphylococcal complement inhibitor; ND, Not Determined; +, Detected; -, Not detected.

*LukS-PV/lukF-PV* and *mupA*-positive MRSA (ST1 and ST8, n=2) were identified in Germany and South Africa (**Tables 2**, **3**). Generally, the *chp*/*sak*/*scn* cluster was detected among strains assigned to CC5, CC22, and CC30 in both countries. The mupR-CC8-MRSA were mainly *sak*/*scn*-positive. Eleven strains (CC8: n=1; CC22: n=7 from Germany; CC22: n=2; CC30: n=1 from South Africa) were negative for the IEC genes (**Tables 2**, **3**).

### 3.3 Detection of antimicrobial and virulence genes in representative mupR-MRSA from Germany and South Africa

Of the 82 mupR-MRSA, 30 strains (South Africa, n=13, Germany, n=17) were selected for WGS. Various antibiotic resistance gene determinants, including chromosomal mutations, were identified. Mutations in the quinolone resistance-determining region, indicating resistance to fluoroquinolones, were identified in 29 strains. Also, antibiotic resistance genes were more widespread among the CC8-MRSA (n=7) than in other lineages (**Supplementary Table 1**). Genes conferring resistance to arsenic (*arsB*, *arsC*, *arsR*) and copper (multicopper oxidase - *mco*) were identified in CC30-MRSA from both countries. These genes were also identified in the *mupA*-positive CC22-MRSA from South Africa, but were absent in five of the six CC22-MRSA from Germany. Moreover, CC8-ST239-MRSA from South Africa possessed the cadmium (*cadC*, *cadD*) and quaternary ammonium (*qacA*) resistance genes, and the mercury resistance (*merA*, *merB*, *merT*) gene operon. These genes were absent in the German CC8-ST239-MRSA strain (**Supplementary Table 2**). In the virulence gene detection, CC30-MRSA from both countries possessed the collagen adhesin (*cna*), glycerol ester hydrolase (*geh*), and toxic shock syndrome toxin-1 (*tst*) genes (**Supplementary Table 2**).

**Figure 1:**
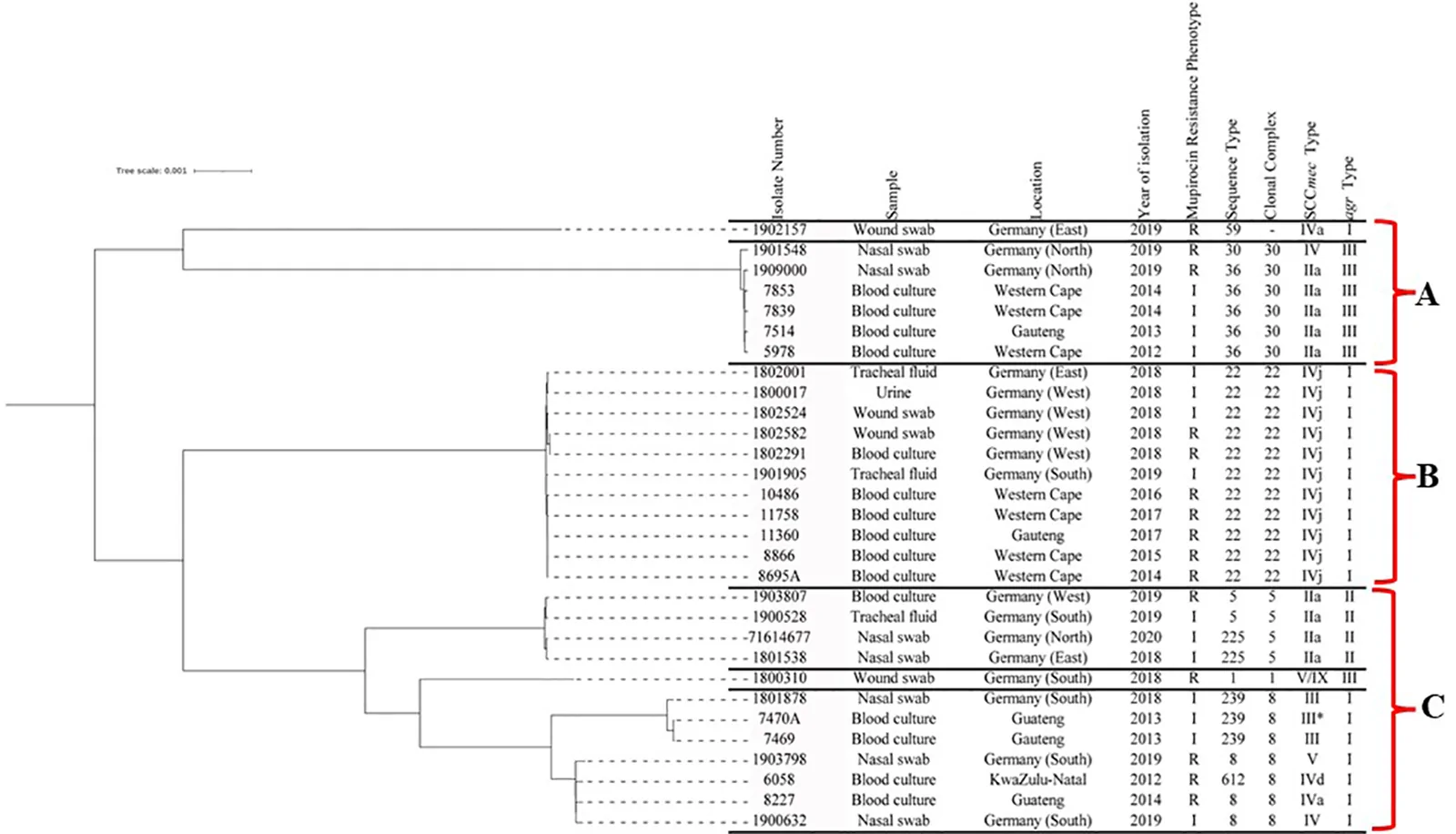
Maximum-likelihood phylogenetic tree of mupR-MRSA from Germany and South Africa. The tree was constructed from the core genome alignment of the whole-genome sequences. Labels A–C denote the phylogenetic clusters identified within the tree. Metadata, mup resistance phenotype, sequence type, clonal complex, SCC*mec* elements, and *ag*r types are provided.

Based on the Maximum-likelihood phylogenetic tree, the representative mupR-MRSA from the two countries were assigned to three main clades without country-specific clusters (**Figure 1**). Group A comprises CC30 and ST59; Group B consists of CC22; and Group C include strains grouped in CC1 and CC8 (**Figure 1**).

### 3.4 SCC*mec* typing

SCC*mec* type IIa, III and IV (IVa, IVd, IVj) were identified among the 30 strains selected for WGS, including one CC1 strain with either an SCC*mec*V or IX. The two SCC*mec* typing tools indicated that one t037-ST239-CC8 strain (7470A) possessed an SCC*mec*XIV element. However, analysis of the SCC*mec*XIV element revealed high structural similarity and gene synteny to the canonical SCC*mec*III element. A notable difference was observed in the cassette chromosome recombinase (*ccr*) gene complex. Whereas classical SCC*mec*III elements typically harbor the *ccrA* and *ccrB* genes, the SCC*mec*III-like element in 7470A lacked both *ccrA* and *ccrB*, but instead carried *ccrC* (**Supplementary Figure 1a**). Representative SCC*mec* elements (IIa, III, IV, IVa, IVj, V) carried by the strains in this study are also described in **Supplementary Figure 1b**. SCC*mec*II was mainly identified in CC5 and CC30 strains, while SCC*mec*IVj element was detected in all CC22 strains from both countries. Diverse SCC*mec* (III, IV, V) elements were observed among CC8 strains (**Figure 1**).

### 3.5 Phylogenomic context of ST8 and ST22 strains

All ST8 strains in this study belonged to the USA300 lineage. The 8227-strain from South Africa clustered within the canonical USA300 clade and exhibited the closest relationship to the USA300 reference strain FPR3757 among the ST8 genomes analyzed (**Supplementary Figure 2a**). Furthermore, all ST22 strains belonged to the EMRSA-15 lineage. Five of the seven ST22 strains (10486, 11758, 11360, 8866, 8695A) from South Africa were more related to each other than to reference ST22 strains, and clustered quite far apart from the remaining two strains from Germany (1802291 and 1802582) (**Supplementary Figure 2b**).

### 3.6 Plasmid analysis

The comparison of plasmids in *mupA*-positive MRSA from this study with the reference *S. aureus* plasmid pPR9 is described in **Supplementary Figures 3a, b**. Based on MOBSCAN, two main plasmid groups were identified. The first group were largely assigned to *rep_cluster_1142* and *rep_cluster_1142*/*rep_cluster_1281*. They possessed the relaxase (MOBQ) genes and the mating pair formation system (MPF_T) (**Table 4**). The second group were generally classified as *rep_cluster_1017*, *rep_cluster_1215*, and *rep_cluster_2214* and possessed the mobilization protein V (MOBV) genes (**Table 4**).

**Table 4:** Characterization of *mupA*-positive plasmids from representative MRSA from Germany and South Africa.

Strain code	ST	CC	Plasmid code	Plasmid size (bp)	% similarity with pPR9	GC content	Replicon type	Relaxase types	Mating pair formation system	Antimicrobial genes on a plasmid	Biocide and heavy metal resistance genes on a plasmid
8227	ST8	CC8	Contig2	37480	74.049	0.287	*rep_cluster_1017* *rep_cluster_1230*	MOBQ	MPF_T	*mupA*	–
			Contig3	38188	75.610	0.286	*rep_cluster_1142* *rep_cluster_1230*	MOBQ	MPF_T	*mupA*	–
6058	ST612	CC8	Contig4	15403	9.756	0.289	*rep_cluster_765*	–	–	*msr(A), mupA*	–
8695A	ST22	CC22	Contig2	44065	19.512	0.296	*rep_cluster_1017 rep_cluster_1215 rep_cluster_2214*	MOBV	–	*blaI, blaR1, blaZ, mupA*	*arsB, arsC, arsR, mco*
8866	ST22	CC22	Contig2	43820	19.512	0.295	*rep_cluster_1017 rep_cluster_1215 rep_cluster_2214*	MOBV	–	*blaI, blaRI, blaZ, mupA*	*arsB, arsC, arsR, mco*
10486	ST22	CC22	Contig2	43849	19.512	0.295	*rep_cluster_1017 rep_cluster_1215 rep_cluster_2214*	MOBV	–	*blaR1, blaZ, mupA*	*arsB, arsC, arsR, mco*
11360	ST22	CC22	Contig2	44052	19.512	0.296	*rep_cluster_1017 rep_cluster_1215 rep_cluster_2214*	MOBV	–	*blaI, blaR1, blaZ, mupA*	*arsB, arsC, arsR, mco*
11758	ST22	CC22	Contig2	43814	19.512	0.295	*rep_cluster_1017 rep_cluster_1215 rep_cluster_2214*	MOBV	–	*blaI, blaR1, blaZ, mupA*	*arsB, arsC, arsR, mco*
			Contig3	41213	19.512	0.295	*rep_cluster_1017 rep_cluster_1215*	–	–	*blaI, blaR1, blaZ, mupA*	*arsR, mco*
1800310	ST1	CC1	Contig2	28001	19.512	0.284	*rep_cluster_1733*, *rep_cluster_2214*	MOBV	–	*blaI, blaZ, mupA*	*cadD, qacA, qacR*
1903807	ST5	CC5	Contig3	38590	73.170	0.287	*rep_cluster_1142*	MOBQ	MPF_T	*aac(6’)-Ie/aph(2’’)-Ia, mupA*	–
1903798	ST8	CC8	Contig2	49836	80.488	0.285	*rep_cluster_1142*	MOBQ	MPF_T	*aac(6’)-Ie/aph(2’’)-Ia, mupA*	–
1802291	ST22	CC22	Contig2	42832	80.488	0.287	*rep_cluster_1142* *rep_cluster_1281*	MOBQ	MPF_T	*aac(6’)-Ie/aph(2’’)-Ia*, *mupA*	*qacC*
1802582	ST22	CC22	Contig2	42809	74.048	0.287	*rep_cluster_1142* *rep_cluster_1281*	MOBQ	MPF_T	*aac(6’)-Ie/aph(2’’)-Ia, mupA*	*qacC*
1901548	ST30	CC30	Contig2	38203	75.610	0.286	*rep_cluster_1142*	MOBQ	MPF_T	*aac(6’)-Ie/aph(2’’)-Ia, mupA*	–
1909000	ST36	CC30	Contig2	43063	53.658	0.277	*rep_cluster_1142*	MOBQ	–	*blaI, blaR1, blaZ, mupA*	–
1902157	ST59	–	Contig2	46300	80.488	0.294	*rep_cluster_1018* *rep_cluster_1142*	MOBQ, MOBQ, MOBV	MPF_T	*aac(6’)-Ie/aph(2’’)-Ia, aadD1, mupA*	–

**Figure 2:**
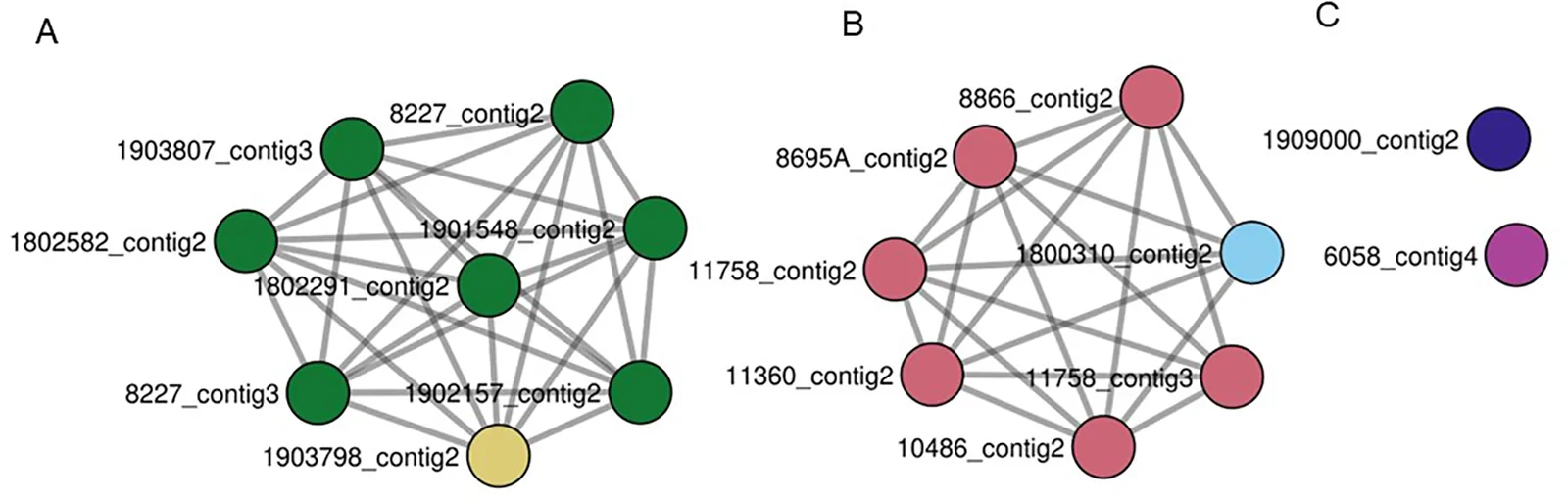
Cluster network of *mupA* gene-carrying putative plasmids for 15 sequenced MRSA genomes. Plasmid communities and sub-communities are assigned based on the plasmid containment distance (threshold = 0.3) and double-cut-join-indel distances (threshold = 3). Plasmid sub-communities are indicated by different node colours within each plasmid network.

**Figure 3:**
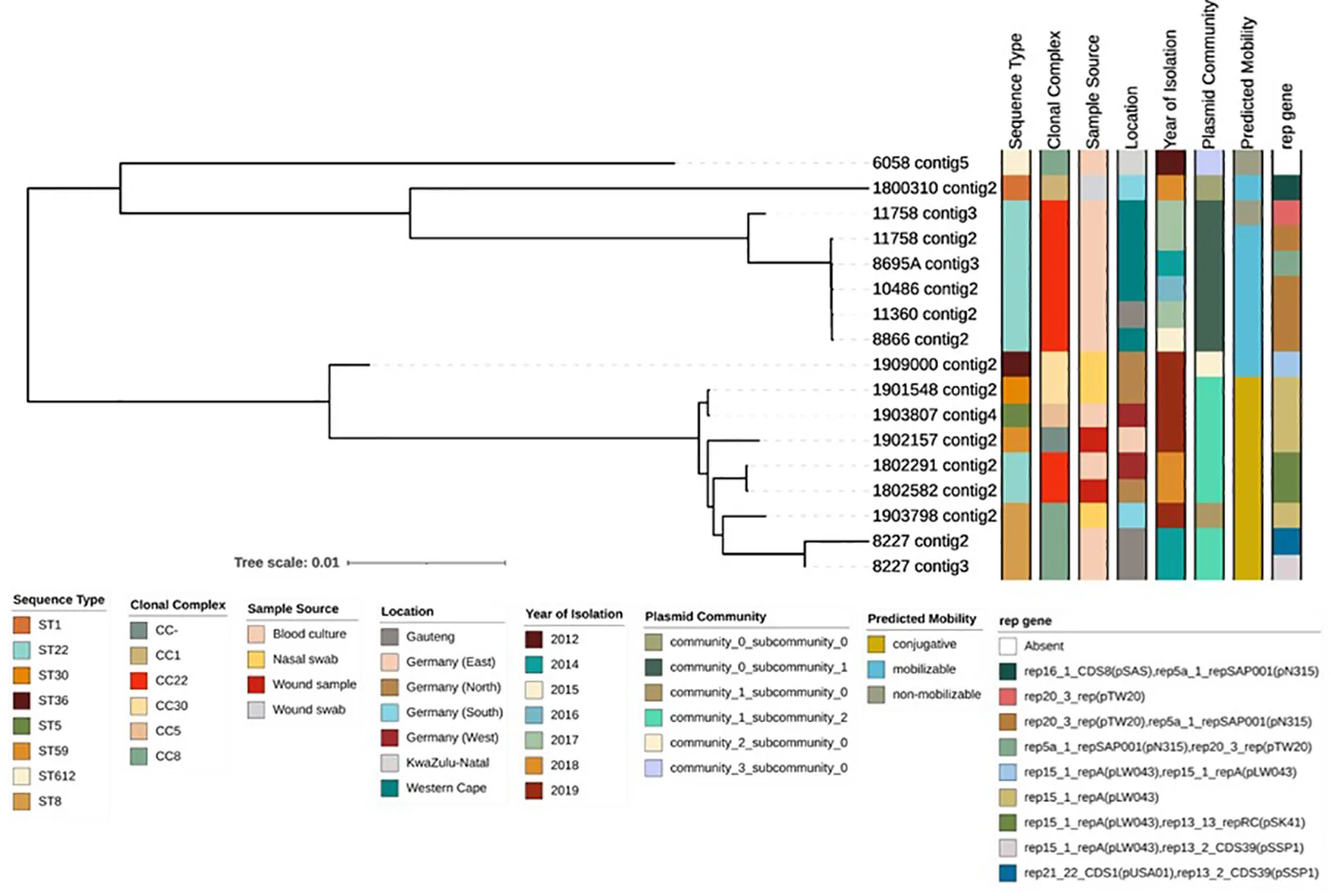
Midpoint-rooted phylogeny of *mupA* gene-carrying contigs extracted from genomes of 15 MRSA strains based on mash distances, accompanied by relevant metadata. Different sequence types, clonal complexes, plasmid community, replication (*rep*) types and source are represented in different colours and intensities.

Using Pling, *mupA*-carrying putative plasmids from the strains from Germany and South Africa were classified into two main plasmid communities, with two additional singleton plasmids (**Figure 2**). The singleton plasmids comprised a non-mobilizable plasmid recovered from a CC8-MRSA isolated from a blood culture in South Africa, and a mobilizable plasmid recovered from a CC30-MRSA from a nasal swab in Germany (**Figure 3**).

**Figure 4:**
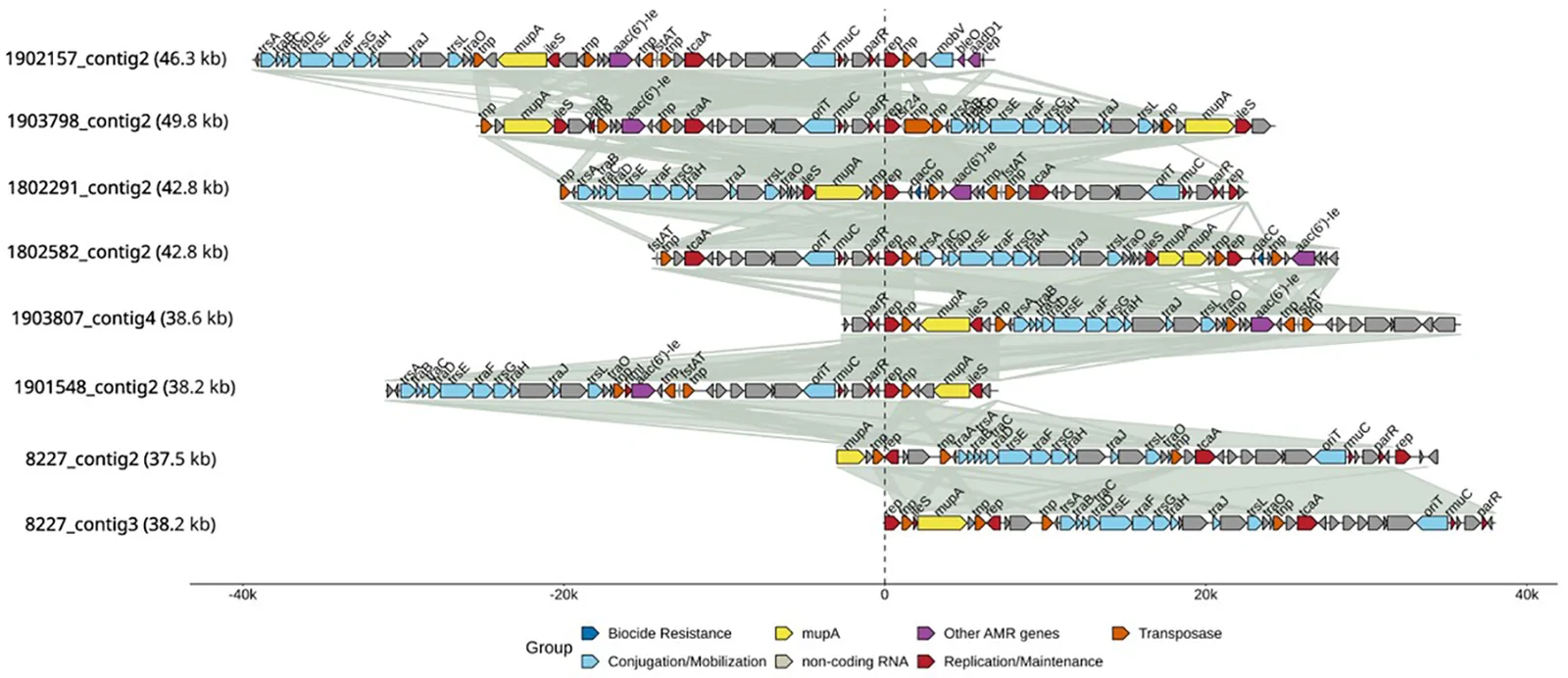
Linear genetic maps of *mupA*-carrying putative plasmids belonging to one of the two large communities (comprising eight putative plasmids, see **Figure 2a**). Yellow arrows represent the *mupA* gene. Grey arrows represent other antimicrobial resistance genes. Red arrows represent replication genes.

The first community (**Figures 2a**, **4**) comprised six of eight plasmids co-harboring at least one aminoglycoside resistance (*aac(6’)-Ie/aph(2’’)-Ia* or *aadD1*) gene. Apart from *qacC* (present in two plasmids), biocide and heavy-metal resistance genes were largely absent. These were conjugative plasmids, identified across diverse CCs (CC5, CC8, CC22, CC30, ST59), and recovered from multiple clinical sources, predominantly in Germany. All conjugative plasmids belonged to the pSK41/pGO1 family and the *rep* family rep15 (**Figure 3**).

**Figure 5:**
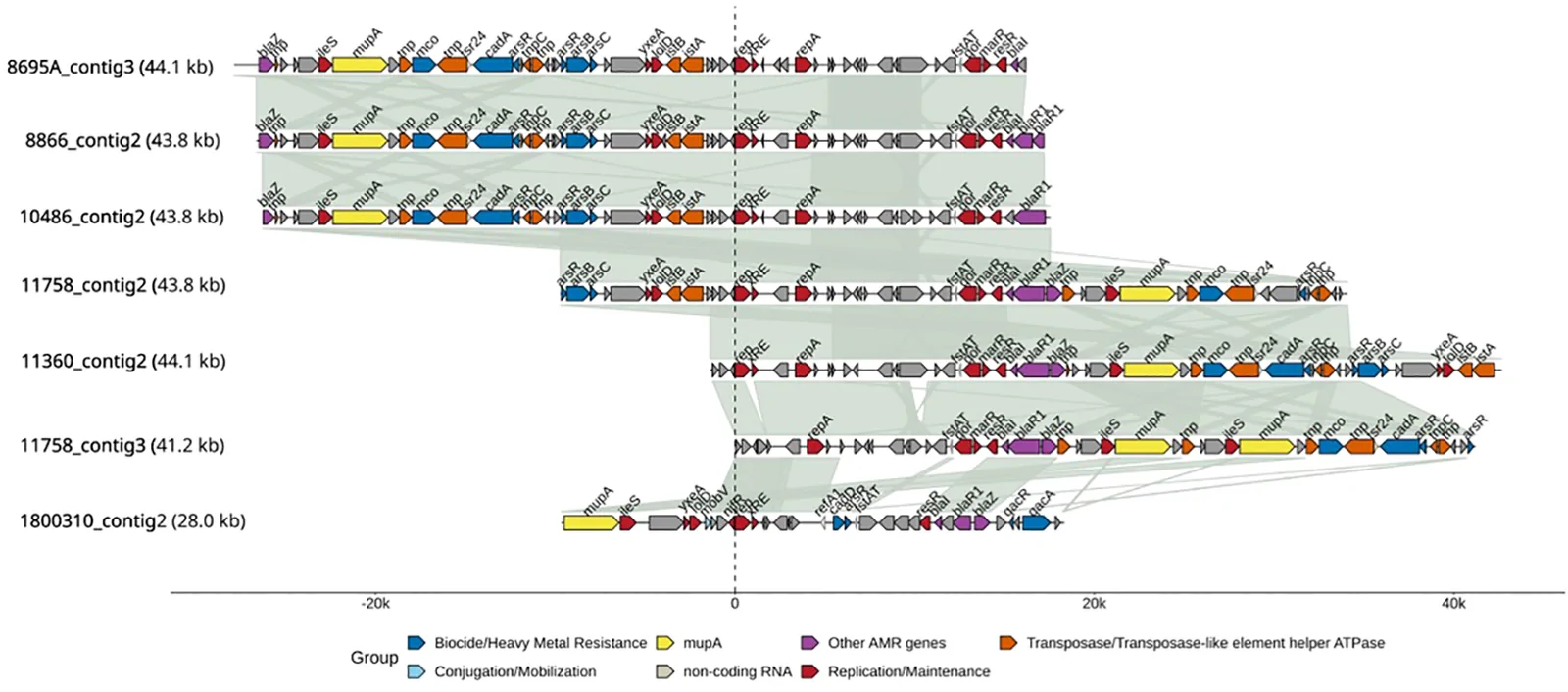
Linear genetic maps of *mupA*-carrying putative plasmids belonging to one of the two large communities (comprising seven putative plasmids, see **Figure 2b**). Yellow arrows represent the *mupA* gene. Gray arrows represent other antimicrobial resistance genes. Blue arrows represent biocide and heavy metal genes. Red arrows represent replication genes.

The second community (**Figures 2b**, **5**) comprises seven *mupA*-carrying plasmids with a broader repertoire of biocide/heavy-metal resistance (*arsR*, *arsB*, *arsC, cadD, mco*, *qacA*) and beta-lactam resistance (*blaI*, *blaR1*, *blaZ*) genes. Most plasmids in this group were mobilizable and mainly recovered from CC22-MRSA isolated from blood cultures in South Africa (**Figure 3**). Mobilizable plasmids from CC22 strains belonged to the replicon families rep5a (n = 1) and rep20 (n = 4).

**Figure 6:**
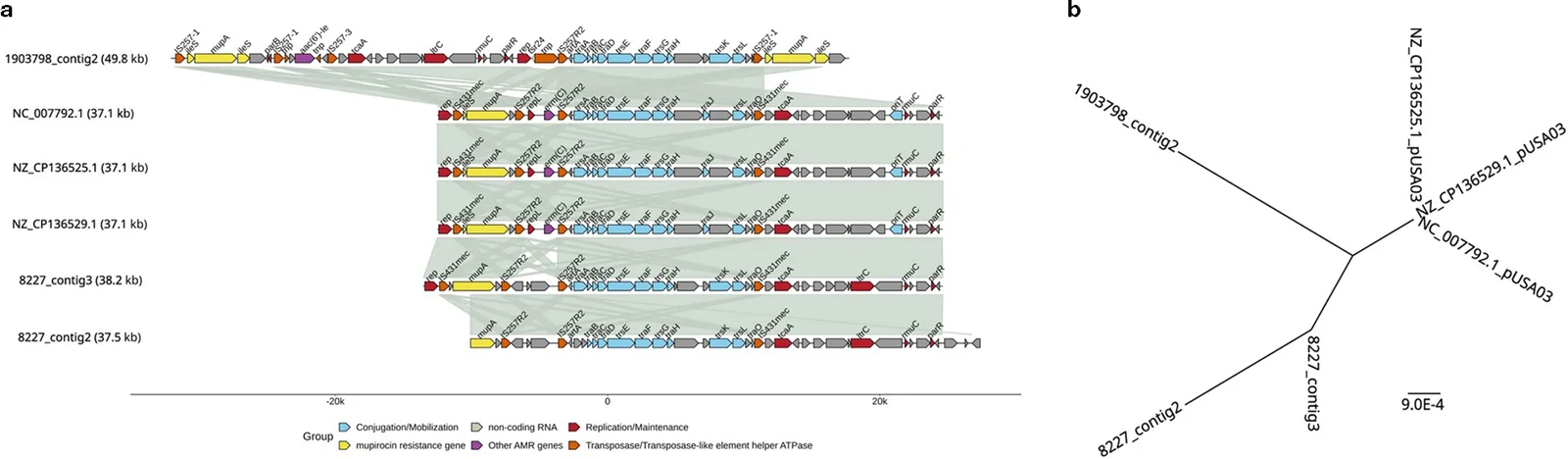
**(A)** Comparison of linear genetic maps of *mupA*-carrying putative plamids of ST8 strains from this study with previously reported USA300 lineages. Yellow arrows represent the *mupA* gene. Red arrows represent replication genes. **(B)** Synteny and distance-based analyses of mupA plasmids of ST8 strains from this study with USA300-associated lineages.

Plasmid comparison analyses demonstrated that at least one of the *mupA*-carrying plasmids identified in one ST8 strain (8227) from South Africa was highly similar in structure to plasmids previously reported in the USA300 lineage (**Figure 6a**). Across synteny and distance-based analyses, this ST8 plasmid clustered closely with USA300-associated *mupA* plasmids (**Figure 6b**).

## 4 Discussion

We investigated the genomic and plasmid characteristics of mupR-MRSA in South Africa and Germany. The study revealed a diverse mupR-MRSA population structure and highlighted the potential for horizontal gene transfer among MRSA lineages in the two countries. MupR-MRSA was identified in various CCs, including CC1, CC5, CC8, CC22, CC30, and ST59 in Germany, and CC8, CC22, and CC30 in South Africa. The broader strain collection from Germany, compared with only blood culture strains from South Africa, and the sampling period may explain the differences in population structure observed between the two countries. Several of these lineages have also been reported in other regions worldwide. For instance, MupR-MRSA has been associated with ST764 in China (Guo et al., 2023), CC8 in India (Abimanyu et al., 2012) and Malaysia (Ghasemzadeh-Moghaddam et al., 2014), CC5, CC8, CC15, and CC22 in Iran (Goudarzi et al., 2018), and CC22 in Portugal (Conceição et al., 2021). Additional reports have described mupR-MRSA in CC8, CC22, and CC45 in Singapore (Hon et al., 2014), CC22 and ST72 in Sweden (Vestberg et al., 2024), CC8 and CC30 in South Africa (Abdulgader et al., 2020), CC8 and CC30 in the United Kingdom (Hughes et al., 2015), and CC5 and CC8 in the United States of America (Virgillio et al., 2025). Collectively, these findings indicate that mupR-MRSA is distributed across diverse genetic backgrounds and resistance has likely arisen through both independent evolutionary events and horizontal gene transfer, possibly mediated by plasmids carrying *mupA*. Additionally, the detection of various SCC*mec* types (IIa, III, IVa, IVj, and V) among the strains in our study supports the idea of multiple evolutionary origins and that mupirocin resistance is not limited to any specific SCC*mec* element. Apart from CC8, MRSA in the different CCs carried the same SCC*mec* element. Notably, all mupR-MRSA assigned to CC22 in both countries possessed the SCC*mec*IVj element. This reflects the global dissemination of a highly successful, hospital-associated clone. The conservation of this SCC*mec* subtype across continents also suggests clonal expansion and intercontinental transmission of a stable genetic background.

ST239-CC8-MRSA-III is one of the oldest pandemic MRSA lineages reported globally, and three major clades (Eurasian, South-East Asian, European/Australian) have been described (Monecke et al., 2018). SCC*mec* typing indicated that one of the ST239-CC8 MRSA strains (7470A) from South Africa possessed the type XIV element. However, further investigation of this element revealed that it was similar to the type III element, but differed with the type 5 *ccr* complex instead of the *ccrA* and *ccrB* (**Supplementary Figure 1a**). This observation represents a novel combination of lineage and the *ccr* complex, underscoring the ongoing evolution and recombination of SCC*mec* elements within local MRSA populations (Monecke et al., 2011). Furthermore, one of the representative ST239-CC8-MRSA from South Africa possessed an SCC*mec*III element carrying both cadmium and mercury resistance genes, consistent with the characterized ccr3–*mec* complex of type III SCC*mec* (Hanssen and Sollid, 2006). However, these genes were absent in another ST239-CC8 MRSA with the same genetic background from Germany, supporting previous reports of regional variation and possible loss of heavy-metal resistance cassettes in SCC*mec* elements (Monecke et al., 2016, 2018).

CC30-MRSA is a pandemic lineage comprising the EMRSA-16 clone (ST36-MRSA-II) prevalent in the United Kingdom and Ireland, and the Southwest Pacific clone (ST30-MRSA-IV), in the Oceanic region (McAdam et al., 2012; Esteves et al., 2025). The *tst* gene encodes toxic shock syndrome toxin-1 (TSST-1), a superantigen responsible for toxic shock syndrome. It is carried on mobile genetic elements such as the staphylococcal pathogenicity island (SaPI) and is commonly found in CC30 lineages (Lindsay et al., 2006). TSST-1-producing *S. aureus* strains are implicated in severe clinical outcomes, particularly in MRSA infections (Touaitia et al., 2025). MupR-CC30-MRSA, with the *tst* gene, was identified in both countries. Moreover, all the representative mupR-CC30-MRSA strains possessed heavy metal (*arsB, arsC, arsR*) and copper (*mco*) resistance gene determinants. This occurrence suggests the accumulation of multiple adaptive traits of selective pressures from antimicrobial and environmental exposures. The mupR-CC30-MRSA strains with these genes from both countries are of public health concern. This combination may enhance survival in both hospital and environmental reservoirs, complicate infection control, and increase the risk of severe disease.

Plasmids often carry genes that enhance bacterial survival, including those conferring resistance to antibiotics, heavy metals, and antiseptics, thereby encouraging horizontal dissemination between unrelated lineages (McCarthy and Lindsay, 2012). The acquired plasmid-encoded *mupA* typically mediates HmupR and resides on mobile plasmids (Gilbart et al., 1993; Patel et al., 2009). In this study, strains exhibiting HmupR carried the *mupA* gene on plasmids across various lineages. Although HmupR was observed in different clonal backgrounds in this study, it was identified mainly in CC8 and CC22 in both countries. Notably, molecular surveillance studies have reported an association between *mupA* carriage and specific clonal backgrounds: CC22 (the EMRSA-15 lineage) and CC8 (including ST8/USA300-related lineages) (Pérez-Roth et al., 2006; Monecke et al., 2017; Lee et al., 2022; Virgillio et al., 2025). These studies suggest that these clones are important reservoirs and vectors for plasmid-mediated HmupR in different geographic settings. Our study observed that the t008-ST8 strain from South Africa was closely related to the USA300 reference strain FPR3757. Plasmid analyses also revealed its high structural similarity and close clustering with previously described USA300-associated plasmids (**Figure 6a, b**). These observations suggest the circulation of a highly conserved mobile genetic element (MBE) within the CC8 lineage. It also indicates that the dissemination of mupirocin resistance may also be driven by the spread of successful MBE across related *S. aureus* populations, underscoring the importance of monitoring MBE in bacterial populations.

Plasmid analysis detected the *qacC* gene in three representative *mupA*-positive German MRSA (**Supplementary Table 2**). This suggests that selective pressure is exerted by the use of quaternary ammonium compounds and other cationic biocides in healthcare settings. The detection of the metal (copper) resistance (*mco*) gene in all the representative CC22-MRSA from South Africa (**Supplementary Table 2**) also implies a potential exposure to metal contaminants. These exposures might influence the selection of these resistance genes. The clustering of the *mupA* plasmids from South African MRSA into a distinct clade in the phylogenetic tree (**Figure 3**) further reinforces the hypothesis of a localized outbreak or clonal dissemination within the country. These findings underscore the critical importance of ongoing surveillance for antibiotic and metal resistance genes, especially in regions exhibiting high prevalence rates. The distinct clustering patterns identified in the phylogenetic tree suggest that tailored, region-specific approaches and genetic insights are adopted in the management of MRSA infections.

The classification of *mupA*-carrying plasmids into four distinct communities, including two singletons (**Figure 2**), reveals substantial diversity in plasmid gene content, mobility, and host backgrounds. The community structure was strongly shaped by differences in resistance gene repertoires, plasmid mobility potential, and host MRSA lineages. These findings signify multiple independent acquisition events rather than clonal spread of a single plasmid lineage. The aminoglycoside gene-rich community is composed entirely of conjugative plasmids and is associated with diverse MRSA sequence types (**Figure 3**, **4**). This community indicates a highly transmissible plasmid lineage that can disseminate across genetically unrelated strains and varied clinical contexts. The apparent scarcity of biocide and heavy metal resistance determinants in this group (apart from sporadic *qacC* occurrence) may suggest either a recent evolutionary focus on antibiotic resistance or adaptation to niches where such genes are less selectively advantageous.

In contrast, the second major community displayed a broader accessory gene repertoire. This included biocide and heavy metal resistance genes as well as beta-lactam resistance determinants, but notably lacked aminoglycoside resistance genes (**Figure 5**). The predominance of mobilizable (rather than conjugative) plasmids within this group (**Figure 3**), combined with its close association with CC22-MRSA from South Africa, suggests a potentially localized evolutionary history. Horizontal transfer is also likely dependent on co-resident conjugative elements. The observed patterns align with previous findings that *mupA* plasmids can act as flexible genetic platforms, integrating resistance determinants through recombination and mobilization events (do Carmo Ferreira et al., 2011). However, the clear segregation of aminoglycoside-rich versus beta-lactam/biocide-rich communities suggests selective pressures arising from regional antimicrobial usage patterns and infection control practices. It may also indicate adaptation to different clinical and epidemiological pressures.

Our study has some limitations. The characterized mupR-MRSA and the antibiotic susceptibility observed in this study may not represent the current trend in the two countries. Moreover, the broad genetic diversity of mupR-MRSA in Germany compared with South Africa could be attributed to differences in strain collection and sampling period. Nevertheless, this study demonstrated that mupR-MRSA exists in diverse clonal lineages in two different geographical regions. Plasmid diversity analyses further indicated that *mupA*-carrying plasmids represent a heterogeneous pool and are structured into distinct genetic communities differing in accessory gene content, mobility, and host association.

## 5 Conclusion

This study demonstrated that mupR-MRSA arises in diverse clonal backgrounds across two geographical regions. These patterns highlight multiple independent evolutionary events. Plasmid diversity analyses further indicated that *mupA*-carrying plasmids form a heterogeneous pool, organized into distinct genetic communities that differ in accessory gene content, mobility, and host association. Plasmids show regional clustering, and antibiotic, biocide, and metal resistance determinants co-occur. This highlights the potential influence of local antimicrobial and environmental pressures on plasmid evolution. Overall, these results emphasize the dynamic interplay between clonal background, mobile genetic elements, and selective factors in understanding the epidemiology of mupR-MRSA. Continued genomic surveillance, antimicrobial use and clinical data are crucial for understanding local adaptation, tracking transmission routes, and guiding infection control strategies against mupR-MRSA lineages.

## Data Availability

The original contributions presented in the study are included in the article/**Supplementary Material**. Genome sequences of the representative mupR-MRSA strains were deposited on the NCBI website under Bioproject accession number PRJNA1060627.

## Glossary

BLAST: Basic Local Alignment Search Tool
BRIG: BLAST Ring Image Generator
CC: Clonal Complex
CDS: Coding Sequence
CI: Confidence Interval
DNA: Deoxyribonucleic acid
EUCAST: European Committee on Antimicrobial Susceptibility Testing
HmupR: High-level mupirocin resistance
*ileS*: Isoleucyl-tRNA synthetase gene
iTOL: Interactive Tree of Life
LmupR: Low-level mupirocin resistance
MALDI-TOF MS: Matrix-Assisted Laser Desorption/Ionization Time-of-Flight Mass Spectrometry
MBE: Mobile Genetic Element
MLST: Multilocus Sequence Typing
MRSA: Methicillin-resistant *Staphylococcus aureus*
MupR-MRSA: Mupirocin-resistant methicillin-resistant *Staphylococcus aureus*
NCBI: National Center for Biotechnology Information
NEB: New England Biolabs
NRPS: nonribosomal peptide synthetase
OR: Odds Ratio
PCR: Polymerase Chain Reaction
*S. aureus*: *Staphylococcus aureus*
SCC*mec*: Staphylococcal Cassette Chromosome *mec*
ST: Sequence Type
WGS: Whole Genome Sequencing
