# Supplementary material for: Molecular characterization of mupirocin-resistant MRSA from Germany and South Africa
Source: Front Cell Infect Microbiol. 2026 Aug 11;16:1865925. doi: 10.3389/fcimb.2026.1865925 (PMC13503169; doi:10.3389/fcimb.2026.1865925)
Supplement: Supplementary file 1 [file Table1.docx]

**Supplementary Table 1: List of antibiotic resistance genes in mupR-MRSA from Germany and South Africa**

|  |  |  | Genes and or mutations encoding resistance against | | | | | | | | | | |
| --- | --- | --- | --- | --- | --- | --- | --- | --- | --- | --- | --- | --- | --- |
| Strain ID | ST | CC | MUP | CHL | ERY | FUS | GEN | QUI | RIF | STR | SUPH | TET | TM |
| 1800310 | ST1 | CC1 | *mupA* | - | - | *fusC* | - | - | - | - | - | - | - |
| 1900528 | ST5 | CC5 | *ileS*_V588F | - | *erm(A)* | - | - | *parC*_E84G /*parC*_S80F | - | - | - | - | - |
| 1903807 | ST5 | CC5 | *mupA* | - | *erm(A)* | - | *aac(6')-Ie/aph(2'')-Ia* | *gyrA*_S84L/*parC*_S80F | - | - | - | - | - |
| 1801538 | ST225 | CC5 | *ileS*_V588F | - | *erm(A)* | - | - | *gyrA*_S84L/*parC*_S80F | - | - | - | - | - |
| 71614677 | ST225 | CC5 | *ileS*_V588F | - | *erm(A)* | - | - | *gyrA*_S84L/*parC*_S80F | - | - | - | - | - |
| 8227* | ST8 | CC8 | *mupA* | - | *mph(C)*/*msr(A)* | - | - | *gyrA*_S84L/*parC*_S80F | - | - | - | - | - |
| 7469* | ST239 | CC8 | *ileS*_V588F | *cat* | *ermA* | - | *aac(6')-Ie/aph(2'')-Ia* | *parC*_S80F | - | - | *folP*_F17L  /*folP*_E258EKE | *tetK*/*tetM* | *dfrG* |
| 7470A* | ST239 | CC8 | *ileS*_V588F | *cat* | *ermA* | - | *aac(6')-Ie/aph(2'')-Ia* | *gyrA*_S84L/*parC*_S80F | - | - | *folP*_F17L  /*folP*_E258EKE | *tetK*/*tetM* | *dfrG* |
| 6058* | ST612 | CC8 | *mupA* | *cat* | *msr(A)* | - | *aac(6')-Ie/aph(2'')-Ia* | *parC*_S80Y | *rpoB*_H48N/ *rpoB*_I527M | *str* | *folP*_F17L  /*folP*_E208K | *tetM* | *dfrC* |
| 1900632 | ST8 | CC8 | *ileS*_V588F | - | - | - | - | *gyrA*_S84L/*parC*_E84V/*parC*_S80Y | - | - | - | - | - |
| 1903798 | ST8 | CC8 | *mupA* | - | - | - | *aac(6')-Ie/aph(2'')-Ia* | *parC*_S80Y | - | - | - | *tetK* | *dfrG* |
| 1801878 | ST239 | CC8 | *ileS*_V588F | - | *erm(A)* | - | *aac(6')-Ie/aph(2'')-Ia* | *gyrA*_G106D/*gyrA*_S84L/*parC*_80F | *rpoB*_H81N/  *rpoB*_L466S | - | - | *tetM* | - |
| 8695A* | ST22 | CC22 | *mupA* | - | *erm(C)* | - | - | *gyrA*_S84L/*parC*_E84G /*parC*_S80F | - | - | - | - | - |
| 8866* | ST22 | CC22 | *mupA* | - | *erm(C)* | - | - | *gyrA*_S84L/*parC*_E84G /*parC*_S80F | - | - | - | - | - |
| 10486* | ST22 | CC22 | *mupA* | - | - | - | - | *gyrA*_S84L/*parC*_E84G /*parC*_S80F | - | - | - | - | - |
| 11360* | ST22 | CC22 | *mupA* | - | - | - | - | *parC*_E84G /*parC*_S80F | - | - | - | - | - |
| 11758* | ST22 | CC22 | *mupA*^Ω^ | - | *erm(C)* | - | - | gyrA_S84L/parC_E84G /*parC*_S80F | - | - | - | - | - |
| 1800017 | ST22 | CC22 | *ileS*_V588F | - | - | - | - | *gyrA*_S84L/*parC*_E84K/*parC*_S80F | - | - | - | - | - |
| 1802001 | ST22 | CC22 | *ileS*_V588F | - | *erm(C)* | - | - | *gyrA*_S84L/*parC*_E84K/*parC*_S80F | - | - | - | - | - |
| 1802291 | ST22 | CC22 | *ileS*_V588F/*mupA* | - | - | - | *aac(6')-Ie/aph(2'')-Ia* | *gyrA*_S84L/*parC*_S80F | *rpoB*_R484H | - | - | - | - |
| 1802524 | ST22 | CC22 | *ileS*_V588F | - | - | - | - | *gyrA*_S84L/*parC*_80F | - | - | - | - | - |
| 1802582 | ST22 | CC22 | *ileS*_V588F/*mupA* | - | - | - | *aac(6')-Ie/aph(2'')-Ia* | *gyrA*_S84L/*parC*_80F/*parE*_P585S | *rpoB*_R484H | - | - | - | - |
| 1901905 | ST22 | CC22 | *ileS*_V588F | - | *erm(C)* | - | - | *gyrA*_S84L/*parC*_S80F | - | - | - | - | - |
| 7514* | ST36 | CC30 | *ileS*_V588F | - | *erm(A)* | - | - | *gyrA*_S84L/*parC*_E84G /*parC*_S80F | *rpoB*_A477D | - | - | - | - |
| 5978* | ST36 | CC30 | *ileS*_V588F | - | *erm(A)* | - | - | *gyrA*_S84L/*parC*_S80F | - | - | - | - | - |
| 7839* | ST36 | CC30 | *ileS*_V588F | - | *erm(A)* | - | - | *gyrA*_S84L/*parC*_S80F | - | - | - | - | - |
| 7853* | ST36 | CC30 | *ileS*_V588F | - | *erm(A)* | - | - | *gyrA*_S84L/*parC*_S80F | - | - | - | - | - |
| 1901548 | ST30 | CC30 | *mupA* | - | - | - | *aac(6')-Ie/aph(2'')-Ia* | - | - | - | - | - | - |
| 1909000 | ST36 | CC30 | *mupA* | - | *erm(A)* | - | - | *gyrA*_S84L/*parC*_E84V/*parC*_S80F | - | - | - | - | - |
| 1902157 | ST59 | - | *mupA* | - | - | *fusA* _H457Y | *aac(6')-Ie/aph(2'')-Ia* | *gyrA*_E88G/*parC*_S80F | - | - | - | - | - |

*: Strains from South Africa; ST: Sequence Type; CC: Clonal Complex; MUP: Mupirocin; GEN: Gentamicin; ERY: Erythromycin; QUI: Quinolones; RIF: Rifampicin; TM: Trimethoprim; SUP: Sulfamethoxazole; FUS: Fusidic acid; STR: Streptomycin; CHL: Chloramphenicol; TET: Tetracycline; -, not detected

**Supplementary Table 2: List of genetic determinants encoding resistance to biocide, heavy metal, quaternary ammonium compounds and virulence genes in mupR-MRSA from Germany and South Africa**

|  |  |  |  | Genes encoding resistance to biocide, heavy metal, quaternary ammonium compounds and virulence determinants | | | | | | | | | | | | | | | | | | | | |
| --- | --- | --- | --- | --- | --- | --- | --- | --- | --- | --- | --- | --- | --- | --- | --- | --- | --- | --- | --- | --- | --- | --- | --- | --- |
| Strain ID | ST | CC | MUP | *arsB* | *arsC* | *arsR* | *cadC* | *cadD* | *mco* | *merA* | *merB* | *merT* | *qacA* | *qacC* | *qacR* | *clfA* | *cna* | *fnbA* | *geh* | *lukD* | PVL | *sed* | *seh* | *tst* |
| 1800310 | ST1 | CC1 | *mupA* | - | - | - | - | + | - | - | - | - | + | - | + | + | + | + | + | + | + | - | + | - |
| 1900528 | ST5 | CC5 | *ileS*_V588F | - | - | - | - | - | - | - | - | - | - | - | - | - | - | - | + | + | - | - | - | - |
| 1903807 | ST5 | CC5 | *mupA* | - | - | - | - | + | - | - | - | - | - | - | - | - | - | - | + | + | - | + | - | - |
| 1801538 | ST225 | CC5 | *ileS*_V588F | - | - | - | - | + | - | - | - | - | - | - | - | - | - | - | + | + | - | + | - | - |
| 71614677 | ST225 | CC5 | *ileS*_V588F | - | - | - | - | + | - | - | - | - | - | - | - | - | - | - | + | + | - | + | - | - |
| 8227* | ST8 | CC8 | *mupA* | - | - | - | - | + | - | - | - | - | - | - | - | - | - | + | + | + | + | - | - | - |
| 7469* | ST239 | CC8 | *ileS*_V588F | - | - | - | + | + | - | + | + | + | + | - | + | - | - | + | - | + | - | - | - | - |
| 7470A* | ST239 | CC8 | *ileS*_V588F | - | - | - | + | + | - | + | + | + | + | - | + | - | - | + | - | + | - | - | - | - |
| 6058* | ST612 | CC8 | *mupA* | - | - | - | - | + | - | - | - | - | - | - | - | - | - | - | - | + | - | - | - | - |
| 1900632 | ST8 | CC8 | *ileS*_V588F | - | - | - | - | + | - | - | - | - | - | - | - | - | - | - | + | + | - | + | - | - |
| 1903798 | ST8 | CC8 | *mupA* | - | - | - | - | - | - | + | + | + | - | + | - | - | - | + | + | + | - | - | - | - |
| 1801878 | ST239 | CC8 | *ileS*_V588F | - | - | - | - | - | - | - | - | - | - | - | - | - | - | - | - | + | - | - | - | - |
| 8695A* | ST22 | CC22 | *mupA* | + | + | + | - | - | + | - | - | - | - | - | - | - | - | + | + | - | - | - | - | - |
| 8866* | ST22 | CC22 | *mupA* | + | + | + | - | - | + | - | - | - | - | - | - | - | - | + | + | - | - | - | - | - |
| 10486* | ST22 | CC22 | *mupA* | + | + | + | - | - | + | - | - | - | - | - | - | - | - | + | + | - | - | - | - | - |
| 11360* | ST22 | CC22 | *mupA* | + | + | + | - | - | + | - | - | - | - | - | - | - | - | + | + | - | - | - | - | - |
| 11758* | ST22 | CC22 | *mupA* | + | + | + | - | - | + | - | - | - | - | - | - | - | - | + | + | - | - | - | - | - |
| 1800017 | ST22 | CC22 | *ileS*_V588F | + | + | + | - | - | + | - | - | - | - | - | - | - | - | - | - | - | - | - | - | - |
| 1802001 | ST22 | CC22 | *ileS*_V588F | - | - | - | - | - | - | - | - | - | - | - | - | - | - | + | + | - | - | - | - | - |
| 1802291 | ST22 | CC22 | *ileS*_V588F /*mupA* | - | - | - | - | - | - | - | - | - | - | + | - | - | - | + | + | - | - | - | - | - |
| 1802524 | ST22 | CC22 | *ileS*_V588F | - | - | - | - | - | - | - | - | - | - | - | - | - | - | + | + | - | - | - | - | - |
| 1802582 | ST22 | CC22 | *ileS*_V588F/*mupA* | - | - | - | - | - | - | - | - | - | - | + | - | - | - | + | + | - | - | - | - | - |
| 1901905 | ST22 | CC22 | *ileS*_V588F | - | - | - | - | - | - | - | - | - | - | - | - | - | - | + | + | - | - | - | - | - |
| 7514* | ST36 | CC30 | *ileS*_V588F | + | + | + | - | - | + | - | - | - | - | - | - | - | + | - | + | - | - | - | - | + |
| 5978* | ST36 | CC30 | *ileS*_V588F | + | + | + | - | - | + | - | - | - | - | - | - | - | + | - | + | - | - | - | - | + |
| 7839* | ST36 | CC30 | *ileS*_V588F | + | + | + | - | - | + | - | - | - | - | - | - | - | + | - | + | - | - | - | - | + |
| 7853* | ST36 | CC30 | *ileS*_V588F | + | + | + | - | - | + | - | - | - | - | - | - | - | + | - | + | - | - | - | - | + |
| 1901548 | ST30 | CC30 | *mupA* | + | + | + | - | - | + | - | - | - | - | - | - | - | + | - | + | - | - | - | - | + |
| 1909000 | ST36 | CC30 | *mupA* | + | + | + | - | - | + | - | - | - | - | - | - | - | + | - | + | - | - | - | - | + |
| 1902157 | ST59 | - | *mupA* | + | + | + | - | + | - | - | - | - | - | - | - | - | - | - | + | - | - | - | - | - |

**. +; detected; -, not detected**

*: Strains from South Africa
